# Supplementary material for: Impact of prolonged infection on SARS-CoV-2 evolution
Source: Microbiol Spectr. 2025 Dec 5;14(1):e02432-25. doi: 10.1128/spectrum.02432-25 (PMC12772307; doi:10.1128/spectrum.02432-25)
Supplement: Figure S1 and Table S1 — Proportion of total iSNVs (%) and intrahost amino acid variants. [file spectrum.02432-25-s0001.docx]

**Supplemental Material of Impact of prolonged infection on SARS-CoV-2 evolution**

Fang Yan^13a^, Qiushi Jin^23a^, Yuanguo Li^3a^, Xuefeng Wang^3^, Chunling Dong^4^, Xianzhu Xia^3^, Yuwei Gao^3*^, Jie Zhang^4*^, Zhijun Hou^1*^

1. College of Wildlife and Protected Areas, Northeast Forestry University, Harbin, China

2. College of Veterinary Medicine, Northeast Agricultural University, Harbin, China

3. Changchun Veterinary Research Institute, Chinese Academy of Agricultural Sciences, State Key Laboratory of Pathogen and Biosecurity, Key Laboratory of Jilin Province for Zoonosis Prevention and Control, Changchun, China

4. Department of Respiratory Medicine, Second Hospital, Jilin University, Changchun, China

^a^ These authors contributed equally to the work.

^*^ These authors contributed equally to the work.

*Corresponding authors:*
Author Yuwei Gao (dawei1105@foxmail.com)

Author Jie Zhang (doctorzhangj@163.com)

Author Zhijun Hou (houzhijundb@163.com)

**Sequence quality filtering**

Using Trimmomatic (v0.39) for quality control of raw sequencing data, removing low-quality bases and adapter sequences.

Trimmomatic PE -phred33 \

Raw_R1.fastq.gz Raw_R2.fastq.gz \

Filtered_R1.fastq.gz Unpaired_R1.fastq.gz \

Filtered_R2.fastq.gz Unpaired_R2.fastq.gz \

ILLUMINACLIP:TruSeq3-PE.fa:2:30:10 \

LEADING:3 TRAILING:3 SLIDINGWINDOW:4:15 MINLEN:36

**Sequence alignment to reference genome**

Using BWA-MEM (v0.7.17) to align filtered high-quality reads to the early reference genome of SARS-CoV-2 Wuhan (NC_045512.2)

bwa mem -t 8 -M \

NC_045512.2.fasta \

Filtered_R1.fastq.gz Filtered_R2.fastq.gz \

| samtools view -bS -q 20 -F 4 - > Aligned.bam

**BAM file sorting and deduplication**

Sort the BAM files using Samtools (v1.10) and remove PCR duplicates using Picard (v2.27.5) to avoid interference from duplicate sequences in the calculation of mutation frequency.

samtools sort -@ 8 -o Sorted.bam Aligned.bam

java -jar picard.jar MarkDuplicates \

I=Sorted.bam \

O=Deduped.bam \

M=DuplicateMetrics.txt \

REMOVE_DUPLICATES=true \

ASSUME_SORTED=true

**iSNV identification and filtering**

Using Bcftools (v1.10.2-34) for variant identification, strict filtering criteria were set based on the characteristics of the SARS-CoV-2 genome to ensure that the detected iSNVs have biological significance.

bcftools mpileup -f NC_045512.2.fasta \

-q 30 -Q 20 -d 10000 -x \

Deduped.bam \

| bcftools call -m -v -O v \

| bcftools filter -i 'INFO/DP >= 50 && INFO/AD[1] >= 5 && (INFO/AD[1]/INFO/DP) >= 0.05 && QUAL >= 50' \

-o Final_iSNVs.vcf

**Table S1 The variation density (mutation sites / 1000nt) of all detected iSNVs**

| **Area** | **Reference length（nt）** | **Number of detected iSNVs (count)** | **Mutation density (mutation sites / 1000nt)** | **Proportion of total iSNVs (%)** |
| --- | --- | --- | --- | --- |
| ORF1ab | 21552 | 42 | 1.95 | 53.8 |
| Spike（S） | 3822 | 16 | 4.19 | 20.5 |
| N | 1260 | 8 | 6.35 | 10.3 |
| ORF3a | 828 | 3 | 3.62 | 3.8 |
| M | 669 | 2 | 2.99 | 2.6 |
| ORF6 | 228 | 3 | 13.16 | 3.8 |
| ORF7a | 366 | 1 | 2.73 | 1.3 |
| ORF8 | 366 | 1 | 2.73 | 1.3 |
| E | 228 | 1 | 4.39 | 1.3 |
| Non-coding region | 2200 | 5 | 2.27 | 6.4 |

**Figure S1 Intra-host amino acid variants**

**
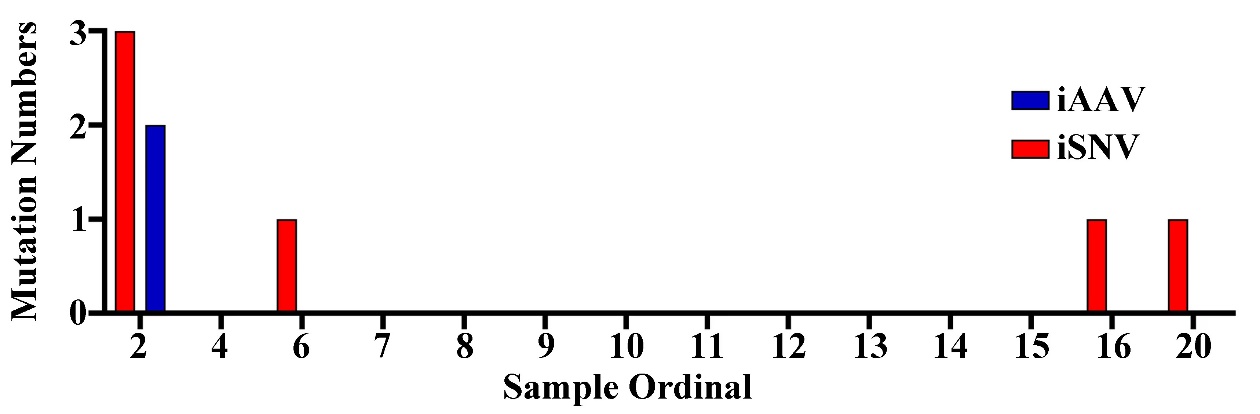
**

Figure S1. iAAV（intra-host amino acid variants）. Novel mutations identified across all samples relative to contemporaneous reference sequences with BA.2. The sample numbers correspond to Table 1, and specific clinical information can be found in Table 1.
